# Full-Digital Manni Telescopic Herbst: structural innovation and digital manufacturing

**DOI:** 10.3389/fdmed.2025.1639784

**Published:** 2025-07-07

**Authors:** Andrea Boggio, Mauro Cozzani, Fabrizio Anelli, Giorgio Gastaldi, Antonio Manni

**Affiliations:** ^1^ Department of Dentistry, Vita-Salute San Raffaele University, Milan, Italy; ^2^ Istituto Giuseppe Cozzani, La Spezia, Italy

**Keywords:** Class II, Herbst, digital, functional treatment, manufacturing

## Abstract

**Introduction:**

Skeletal Class II malocclusion, commonly characterized by mandibular retrusion, affects a significant portion of the population and presents challenges in orthodontic correction. The Herbst Appliance has long been used for mandibular advancement, but traditional designs often lead to undesirable dental side effects and mechanical complications. This paper introduces the structural innovation and digital manufacturing of Full Digital Manni Telescopic Herbst (MTH) Appliance, a structurally innovative and digitally manufactured system aimed at enhancing clinical outcomes and reducing treatment failures.

**Materials and equipment:**

Utilizing a fully digital workflow, including CAD/CAM design, intraoral scanning, and additive manufacturing, the MTH appliance integrates a full-coverage mandibular splint, digitally designed and precision-engineered maxillary and mandibular components.

**Results:**

The clinical implementation of the MTH appliance demonstrates excellent fit and high mechanical reliability, reducing lower incisor proclination and offering a proper vertical control. Complications such as debonding or fractures can be significantly reduced and digital reproducibility allows for fast component replacement when needed.

**Discussion and conclusion:**

This design improves skeletal correction by enhancing anchorage, minimizing lower incisor proclination, and allowing precise vertical and sagittal control. The digital workflow not only improves fabrication accuracy and patient comfort but also facilitates easy component replacement and reduced chairside time. The MTH appliance sets a new benchmark in Class II treatment, blending digital precision with biomechanical effectiveness.

## Introduction

Skeletal Class II malocclusion represents one the most prevalent dentofacial conditions, affecting nearly one-third of the North American population (1). Its prevalence among Caucasian orthodontic patients is reported to be as high as 36%–48% (2). The hallmark of this condition is mandibular retrusion, which typically results in a convex facial profile and retrognathic chin appearance (3).

Over the past century, numerous treatment modalities have been developed to address Class II malocclusions, ranging from growth modification appliances during adolescence to orthognathic surgery in severe adult cases. Functional orthopedic devices play a key role during growth phases and are broadly categorized as “Removable Appliances”, such as the Twin Block, Frankel, Activator, and “Fixed Appliances”, such as Forsus and the Mandibular Anterior Repositioning Appliance (MARA). Both types aim to correct intermaxillary discrepancies and reduce overjet (4), but Fixed Appliances are often considered more efficient due to reduced dependency on patient compliance (5).

One of the most established Fixed Functional Appliances is the Herbst Appliance, first introduced by Emil Herbst in 1906. It exerts both skeletal and dentoalveolar effects, including forward repositioning of the mandible, inhibition of maxillary sagittal growth, advancement of the mandibular arch, and distalization of the upper arch (6). While skeletal effects are favorable, dental side effects such as palatal tipping of upper incisors, labial proclination of lower incisors, and uncontrolled movements of posterior teeth are generally undesired. In fact, these side-effects can compromise skeletal outcomes by excessively reducing the overjet, thereby diminishing the space for mandibular advancement (7).

**Figure 1:**
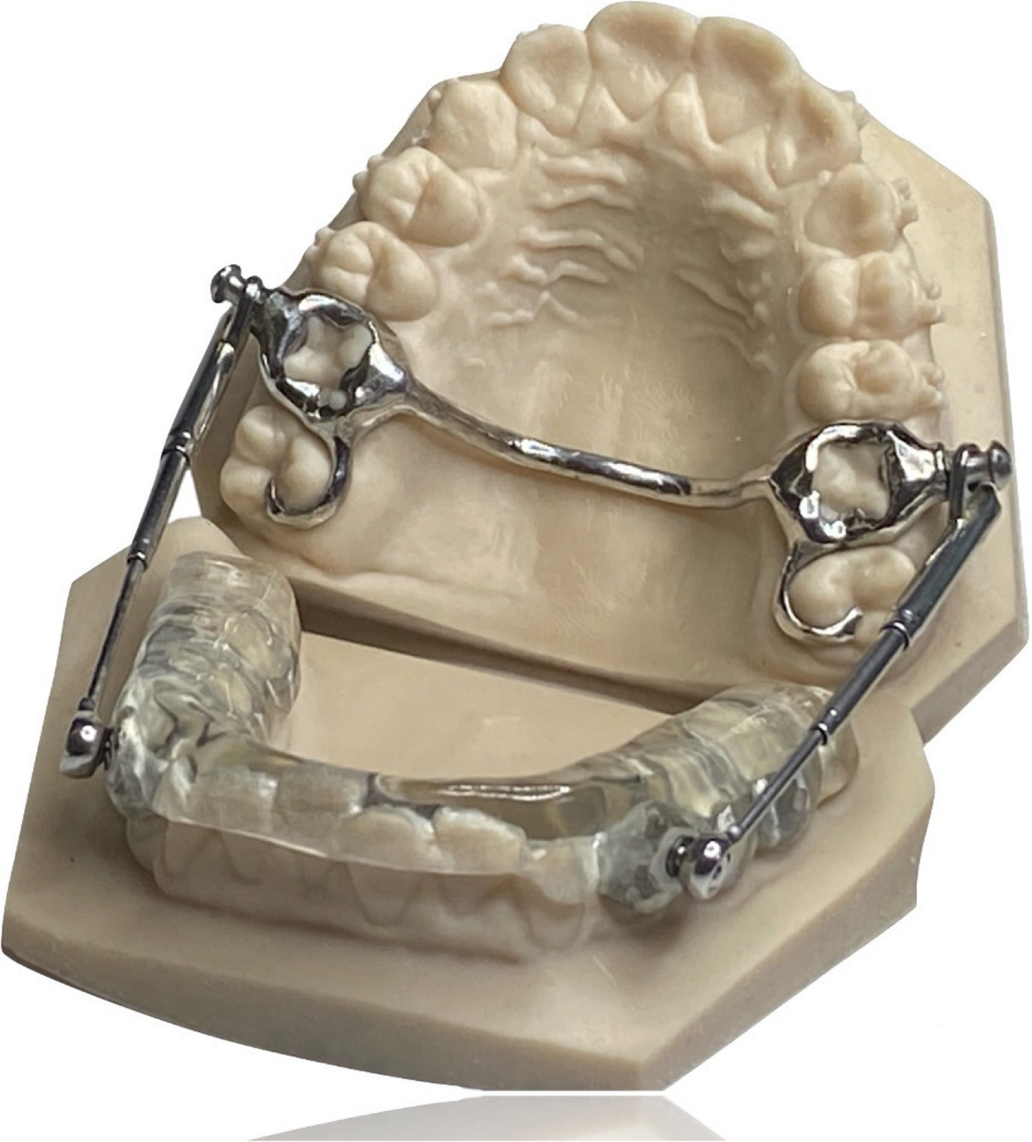
Full-Digital Manni Telescopic Herbst Appliance.

To address these limitations, several modifications of the original Herbst design have been proposed (7–11). One such innovation is the Manni Telescoping Herbst (MTH) Appliance, which incorporates a fixed palatal arch with Rollo-Bands connected to a mandibular acrylic splint through bilateral telescoping rods. These rods permit up to 12° of lateral mandibular movement, while the splint limits proclination of the lower incisors and can be partially removed for improved oral hygiene (12) (Figure 1).

Despite enhancements in design and reliability over previous versions, such as the Hanks Telescoping Herbst and MiniScope, issues such as fractures of molar bands, debonding, splint breakage or soft tissue lesions, can rarely occur, often necessitating repair or full appliance replacement (13). These setbacks increase treatment time, cost, and chairside workload.

Recent advances in digital orthodontics, especially the integration of Computer-Aided Design and Manufacturing (CAD/CAM) systems, 3D printing, and laser-melting technologies, are transforming appliance fabrication, reducing human errors and streamlining production processes. These methods enable the creation of fully customized, highly accurate and resistant, and biomechanically optimized devices using biocompatible materials, furtherly increasing personalization and efficiency.

In this context, the Full Digital Manni Telescopic Herbst Appliance exemplifies the next generation of orthodontic innovation. Its digital protocol employs CAD-CAM design and additive manufacturing to deliver a structurally refined, patient-specific mandibular advancement device. Enhanced anchorage, reduced mechanical complications, and improved clinical efficiency position the Full Digital Herbst MTH as a promising tool in the contemporary treatment of Class II malocclusion. The aim of this paper is to outline the step-by-step digital workflow, design rationale, and clinical advantages of the Full Digital MTH Appliance, setting a new benchmark in the treatment of Class II malocclusion.

## Materials and equipment

### Digital impression and mandibular positioning

The process begins with obtaining high-resolution intraoral scans of the maxillary and mandibular arches using a digital scanner. The esthetic Fränkel maneuver (14) or the Manni's Aesthetic maneuver (15) is recommended to determine the optimal esthetic advancement, without causing excessive strain on the temporomandibular joints or perioral musculature. With the mandible held in advanced sagittal position, the scans capture the occlusal relationship required for appliance construction. The mandible can be stabilized during the impression using a piece of wax placed on the incisors. This digital impression eliminates the need for traditional alginate or PVS impressions, reducing chair time and patient discomfort. Moreover, the digital record serves as a reusable dataset if components need to be replaced or modified during treatment.

### Maxillary component design: digital bands and transpalatal arch

**Figure 2:**
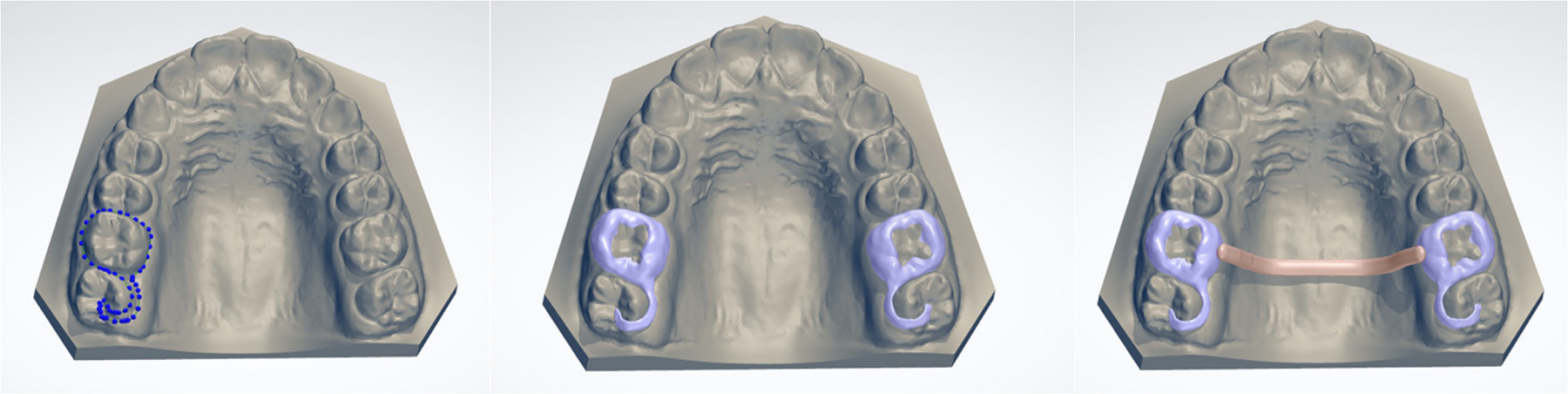
Maxillary component design: digital bands and transpalatal arch.

The design of the maxillary component begins with creating digital custom bands on the upper first molars using the “Create Shell” command in “Appliance Designer CAD software” (3Shape, Copenhagen, Denmark). The bands are set to a thickness of 0.5 mm with a 0.05 mm offset to allow for cement space, and the insertion direction is aligned perpendicular to the occlusal plane. This ensures ease of placement and optimal adhesion. The digital bands are extended distally to incorporate occlusal rests on the second molars, which serve to prevent vertical over-eruption of those teeth during treatment. The transpalatal arch is modeled using a 1.5 mm diameter bar that connects the molar bands while maintaining a 1 mm clearance from the palatal mucosa (Figure 2). This clearance minimizes the risk of soft-tissue irritation or ulceration. Using the same software, virtual tools such as the “Wax Knife” and “Spine Cut commands” are employed to refine the design, smooth edges, and remove any unnecessary projections. The final geometry is unified into a single maxillary component using the “Combine Models” function.

### Mandibular component design: digital bands and STL hinge integration

**Figure 3:**
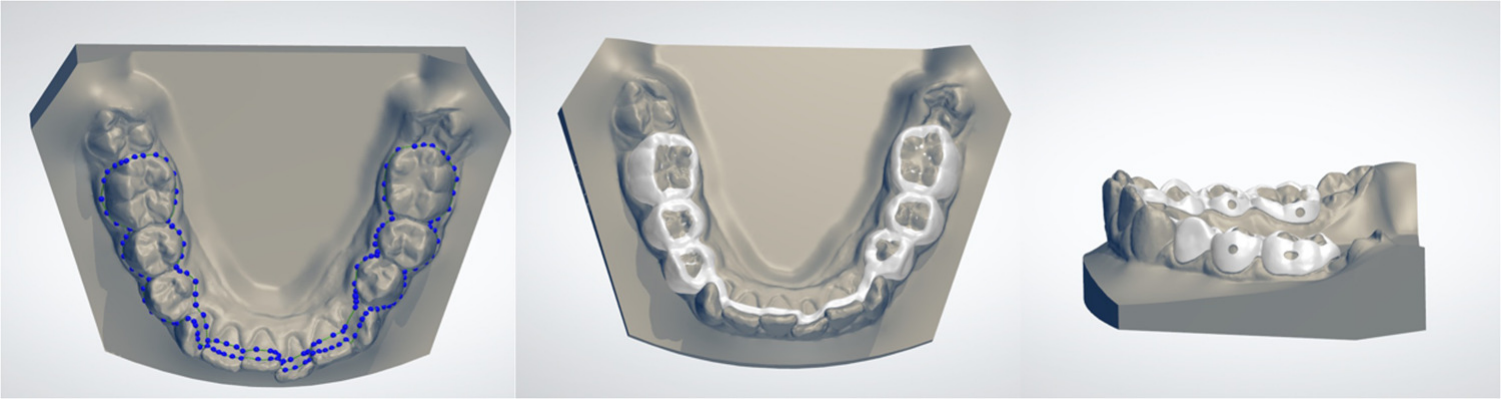
Mandibular component design: digital bands and lower substructure.

**Figure 4:**
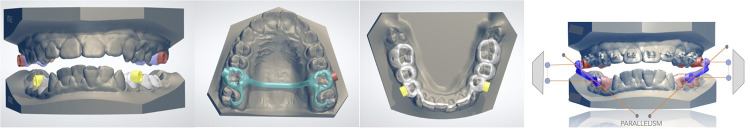
STL hinge integration with virtual parallelometer.

The mandibular segment involves designing bands located on the first and second premolars, with the same shell thickness and offset parameters as the upper bands (Figure 3). In this way a laser-melted cobalt-chromium or titanium substructure is created. Calibrated STL files are used to create hinge nuts, virtual slots that guide the precise placement of the Herbst hinges, with Rhinocheros CAD (Registered trademark of Robert McNeel & Associates, Seattle, WA). Using a digital parallelometer (Figure 4), the parallel alignment between the upper and lower hinge axes can be checked and confirmed; this step is critical for minimizing mechanical stress during function and reducing the risk of breakages. Once the design is verified, the nuts are removed, and the hinges are later laser-welded onto the metal framework.

The hinges (American Orthodontics, Sheboygan, WI) are available in various lengths (18, 21, 24, and 27 mm) to accommodate individual anatomical needs. They are posteriorly beveled to minimize soft tissue irritation and feature a lower hex screw design, allowing up to 12° of lateral mandibular excursion. This controlled freedom of movement provides sufficient rigidity to prevent hinge fractures while also reducing the risk of mucosal trauma often associated with excessive lateral motion (13).

### Mandibular component design: full-coverage lower splint

**Figure 5:**
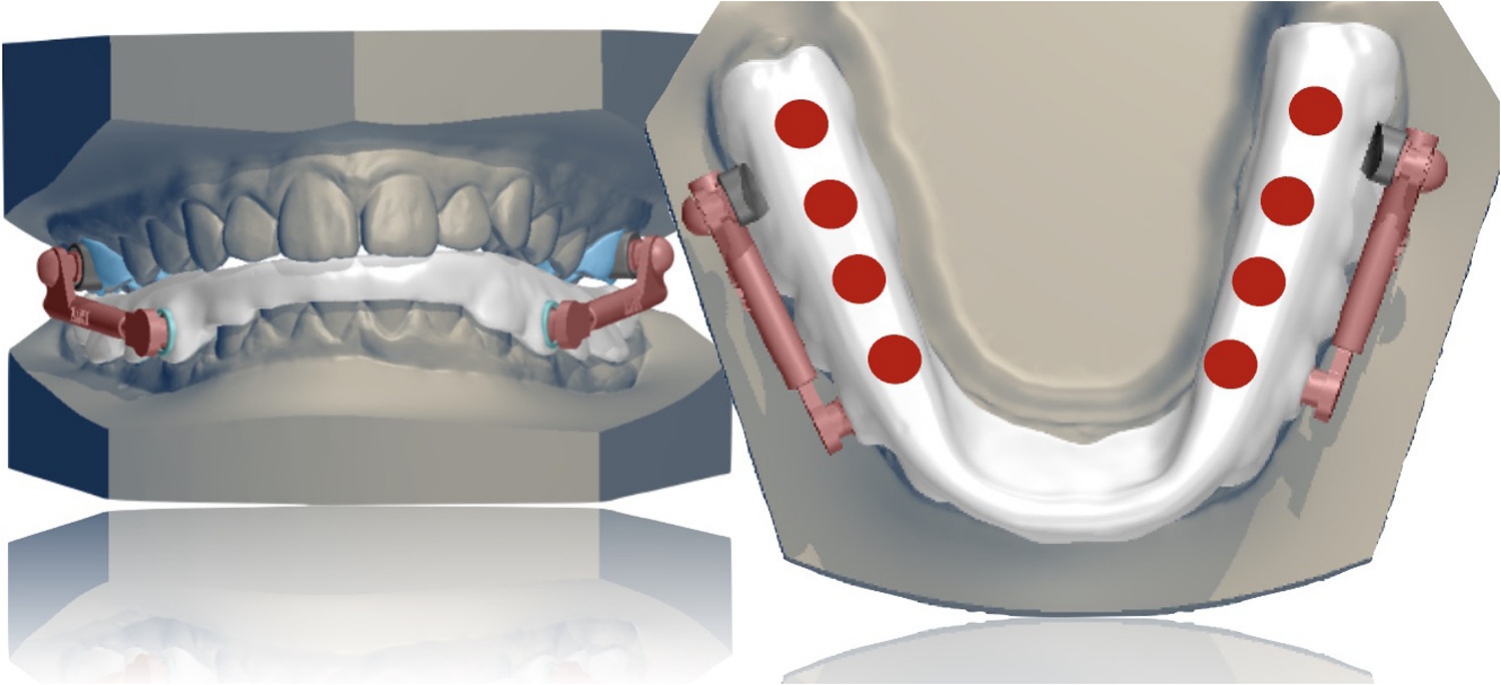
The lower acrylic splint.

The most innovative component of the MTH appliance is its full-coverage mandibular splint, digitally designed using CAD/CAM software and constructed over the laser-melted cobalt-chromium or titanium substructure (Figure 5). The acrylic splint, digitally designed and fabricated using 3D printing with biocompatible resins ensures patient safety and tolerability, while offering biomechanical advantages derived from its full-coverage design and integration with a rigid metallic substructure. This configuration allows for:

• Fracture Resistance: Unlike conventional systems with individual bands, the unified splint structure provides enhanced mechanical stability, significantly reducing the risk of appliance failure.
• Control of Lower Incisor Proclination: A labial resin shield counteracts mesial forces, preserving overjet space and minimizing lower incisor tipping—from approximately 10.5° in conventional Herbst appliances to around 7.5° with the MTH system (12, 16).
• Vertical Dimension Management: Posterior occlusal contact is maintained, making the appliance suitable for hyperdivergent patients, while simultaneously enabling greater sagittal mandibular advancement due to the controlled increase in vertical dimension (17).
• Consolidated Anchorage: The splint itself creates a single lower anchorage unit, which can be blocked using two miniscrews positioned between the second premolars and first molars and connected to the arch by elastic chains from the screws to buttons on buccal surface of lower canines (9).
• Protection of Temporomandibular Structures: By optimizing load distribution and mandibular guidance, the design may help prevent TMJ problems, particularly in high-risk patients (18, 19).

**Figure 6:**
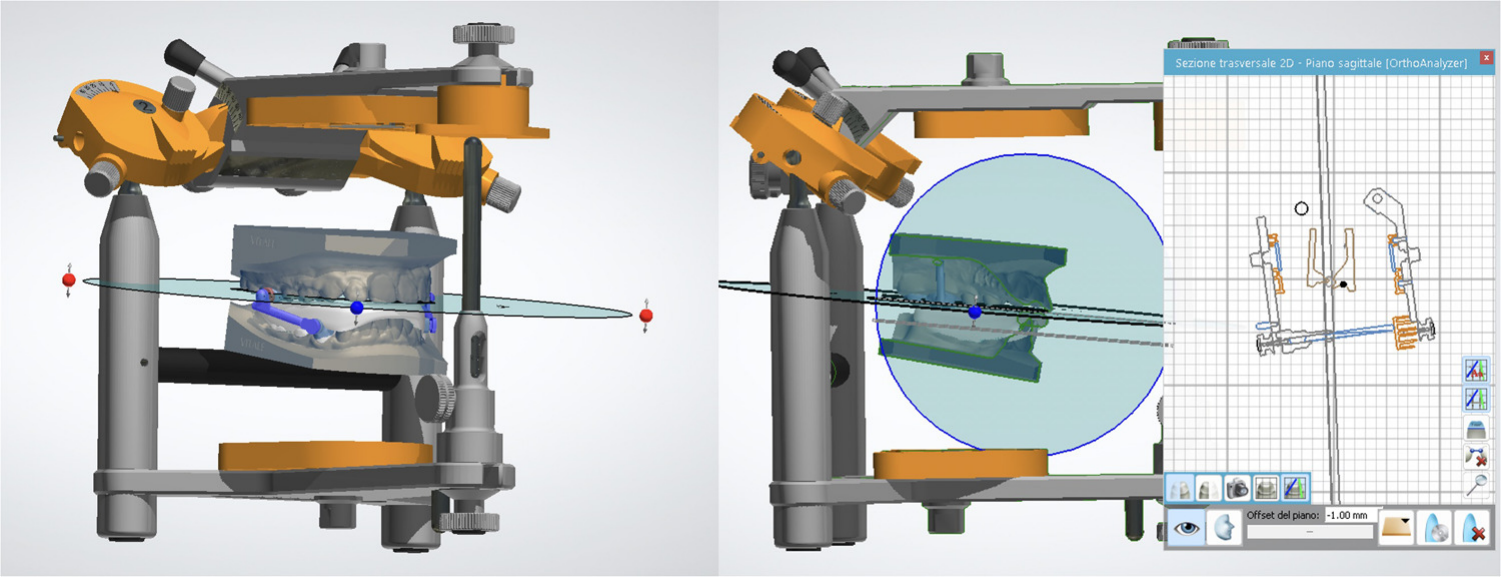
Virtual articulator used during the fabrication of the appliance.

Digital precision plays a key role in the design and effectiveness of the MTH mandibular splint. Through the use of CAD/CAM tools, clinicians can achieve consistent and clinically appropriate splint thickness (approximately 1.5 mm in the molar region) ensuring both structural integrity and patient comfort. This technology also enables highly accurate positioning of occlusal contacts. The virtual articulator tool simulates mandibular movements, allowing for real-time assessment of hinge clearance and splint stability (Figure 6).

When adding skeletal anchorage is required, with a quick simulation, the exact placement of bondable buttons on the buccal surface of lower canines can be planned to avoid interference with the splint and maintaining a 1 mm clearance from the gingival margin. Additionally, this design feature minimizes the risk of interference of elastic ligatures with hinges, thereby enhancing the overall precision of the appliance and contributing to the therapeutic success of the appliance.

### Fabrication and assembly

Following digital design, all components are exported as STL files. The metal parts are produced via selective laser melting using biocompatible cobalt-chrome or titanium alloys. The splint is constructed by layering resin over the metal substructure or with a 3D printer. While fully 3D-printed splints are feasible, in some cases they are not preferred due to increased brittleness and risk of fracture. Instead, hybrid construction using a metal base and resin overlay can be more secure.

Assembly steps include:

• Laser-welding hinges to the band frameworks.
• Bonding the splint to the lower framework using light-cured resin.
• Polishing all surfaces.

### Clinical delivery and final adjustments

**Figure 7:**
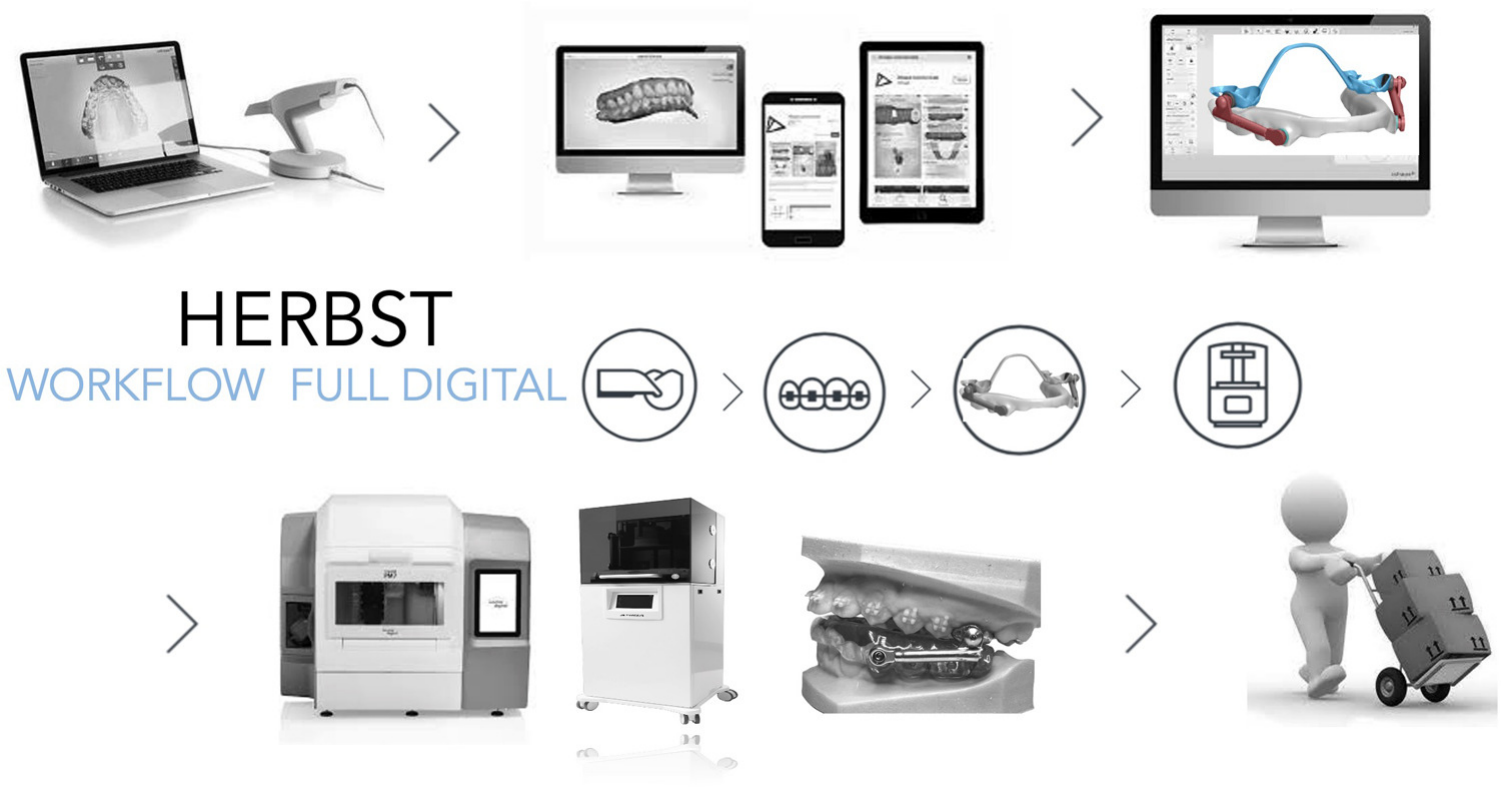
Full-digital workflow of the appliance production.

Prior to delivery, the assembled appliance undergoes a final fit check on the analog articulator to simulate intraoral conditions. Occlusal relationships, hinge movement, and appliance stability, previously simulated with the virtual tool, are then assessed and adjusted as needed (Figure 7).

## Results

The implementation of the Full-Digital Manni Telescopic Herbst (MTH) appliance in clinical practice is going to offer promising outcomes in terms of mechanical performance, treatment precision, and workflow efficiency. The digital workflow enables a complete integration of intraoral scans and CAD/CAM-based appliance fabrication, limiting the need for significant chair-side adjustments.

A high reliability in the parallel alignment of the telescopic hinges can be expected, thanks to the use of the virtual parallelometer during design, while the digitally adjusted splint preserves balanced contact and facilitates mandibular advancement, particularly beneficial in hyperdivergent patients.

**Figure 8:**
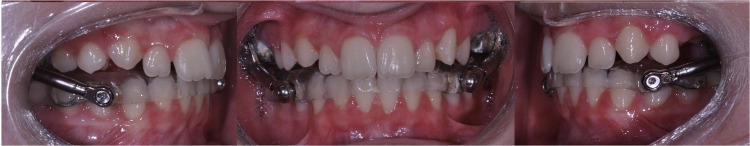
Intraoral images of tache Full-Digital Manni Telescopic Herbst Appliance.

Furthermore, the full-coverage mandibular splint can limit lower incisor proclination (Figure 8). This effect is largely attributable to the acrylic splint, which consolidates the lower dentition into a single anchorage unit, distributing mesial forces and thereby reducing the risk of lower incisor proclination. This principle was confirmed by previous studies, who observed improved incisor control in Herbst appliances employing full mandibular coverage splints (20, 21). Additionally, the splint's structural reinforcement minimized the occurrence of breakages, contributing to treatment continuity and reducing interruptions (13).

Moreover, the option to combine the appliance with virtual simulation of miniscrew placement can be particularly useful in cases requiring enhanced anchorage, limiting interference with appliance components. Despite the complications are limited by such a workflow, in the rare cases of wear or patient-related damage, the digital archive of STL files allows for rapid reprinting of individual components, eliminating the need for new impressions.

## Discussion

The Full-Digital Manni Telescopic Herbst Appliance exemplifies the integration of digital technologies with biomechanics of functional appliances to overcome some limitations related to conventional Class II appliances. By leveraging CAD/CAM design, additive manufacturing, and virtual simulations, this appliance represents a clinically precise and biomechanically optimized alternative to conventional Herbst treatments.

Among the innovations, the key element is the full-coverage mandibular splint, which reduces of fifty per cent the probability of detachment (the appliance is a fixed functional device bonded only in the upper arch) and creates a single lower anchorage unit. This structural innovation not only minimizes the risk of mechanical failure, commonly observed in systems relying on molar bands in both the arches, but also enhances biomechanical control, particularly in limiting lower incisor proclination (12). This is especially relevant considering that flaring of lower incisors is a frequent side-effect of traditional Herbst therapy, which can negatively affect the overall skeletal correction, by prematurely exhausting the overjet (20). The resin shield, supported by its rigid framework, allows to reduce this effect and facilitates greater sagittal mandibular repositioning, offering enhanced efficacy in skeletal Class II correction. If even more anchorage is needed, this system can be perfectly combined with lower miniscrews, whose insertion can be virtually simulated to ensure the absence of any interference with the appliance. The lower inter-radicular TADs are generally inserted bilaterally between the first molar and the second bicuspid or between the first and the second bicuspid, based on bone availability (22). They are connected through elastic chains to buttons bonded on the labial surface of lower canines, significantly reducing dental compensations, such as labial flaring of lower incisors (9, 12).

In addition to sagittal dimension, vertical control is another area where the MTH appliance demonstrates clear clinical benefit. The presence of strong posterior occlusal contacts through digital planning encourages a counterclockwise rotation of the mandible, stimulating a greater advancement of the Pogonion and making the system suitable also for hyperdivergent patients (17, 21).

The ability to simulate mandibular dynamics with the virtual articulator, to align hinge axles using a digital parallelometer, and to fabricate components with micrometric accuracy significantly reduces failures, intraoral adjustments and chairside time (23). Additionally, one of the key advantages of the Full Digital MTH system is its reproducibility. In case of issues, clinicians can reprint or remanufacture individual components from stored STL files without requiring new impressions, with a substantial advantage in terms of clinical efficiency.

Despite these benefits, some limitations must be considered. First, the system's reliance on digital infrastructure necessitates access to digital design tools, as well as familiarity with specific software. Furthermore, while the splint design provides improved mechanical resistance, its hybrid construction—particularly the bond between metal substructure and acrylic overlay—may represent a weak point. In particular, fully 3D-printed resin splints, although possible, may be more susceptible to fatigue and breakages over time.

Another consideration is related to cost: although digital workflows ultimately reduce manufacturing variability and enable rapid component reproduction, the initial investment in technology, training, and software licensing may be significant, if compared with traditional appliances.

## Conclusion

The Manni Telescopic Herbst appliance, especially in its Full-Digital version, represents a clinically relevant step in the evolution of functional orthodontic appliances. By integrating digital scanning, CAD/CAM design, additive manufacturing, and biomechanical optimization, the system provides a high degree of customization, durability, and clinical effectiveness.

Its precise architecture and full-coverage splint offer great control encouraging mandibular advancement and limiting dentoalveolar movements. Moreover, the digital workflow facilitates reproducibility and minimizes chairside adjustments, ultimately improving satisfaction of both clinicians and patients.

As orthodontics continues to embrace digital innovation, systems like the MTH Appliance may probably represents the standard for future appliance design, offering an unparalleled combination of precision, efficiency, and clinical control.

## Data Availability

The original contributions presented in the study are included in the article/Supplementary Material, further inquiries can be directed to the corresponding author.
